# Crystallographic Evaluation of Susceptibility to Intergranular Corrosion in Austenitic Stainless Steel with Various Degrees of Sensitization

**DOI:** 10.3390/ma13030613

**Published:** 2020-01-30

**Authors:** Tomoyuki Fujii, Takaya Furumoto, Keiichiro Tohgo, Yoshinobu Shimamura

**Affiliations:** Department of Mechanical Engineering, Shizuoka University, Hamamatsu 4328561, Japan; furumoto.takaya.15@shizuoka.ac.jp (T.F.); tohgo.keiichiro@shizuoka.ac.jp (K.T.); shimamura.yoshinobu@shizuoka.ac.jp (Y.S.)

**Keywords:** intergranular corrosion, sensitization, austenitic stainless steel, grain boundary, grain boundary structure

## Abstract

This study investigated the susceptibility to intergranular corrosion (IGC) in austenitic stainless steel with various degrees of sensitization (DOSs) from a microstructural viewpoint based on the coincidence site lattice (CSL) model. IGC testing was conducted using oxalic acid and type 304 stainless steel specimens with electrochemical potentiokinetic reactivation (EPR) ratios that varied from 3 to 30%. As a measure of IGC susceptibility, the width of the corroded groove was used. The relationship between IGC susceptibility, grain boundaries (GB) structure, and EPR ratio of the specimens was evaluated. As a result, the IGC susceptibility cannot be characterized using the Σ value, irrespective of the DOS of the specimen. The IGC susceptibility increases with increasing unit cell area of CSL boundaries, which is a measure of the stability of the CSL boundaries, and then levels off. The relationship between the IGC susceptibility and unit cell area is sigmoidal, irrespective of the DOS of the specimen. The sigmoid curve shifts rightward and the upper bound of IGC susceptibility decreases with decreasing DOS of the specimen.

## 1. Introduction

Austenitic stainless steel exhibits superior mechanical properties and corrosion resistance to aqueous, gaseous, and high-temperature environments, and hence is used for pipes in chemical plants and coolant pipes in light water reactors. Machines and structures made of steel are usually assembled by welding, and it is well-known that sensitization of stainless steel occurs during welding. At high temperature, precipitation of chromium carbide at grain boundaries (GBs) occurs in stainless steel, which forms chromium depletion zones in the vicinity of the GBs, resulting in a loss of resistance to intergranular corrosion (IGC) and intergranular stress corrosion cracking [1]. 

In an effort to improve the resistance of stainless steel to IGC, the IGC susceptibility has been investigated from electrochemical and microstructural viewpoints. Xin et al. [2] investigated the IGC susceptibility in TIG-welded 316LN stainless steel by the double-loop electrochemical potentiokinetic reactivation (DL-EPR) technique, and found that a welded area exhibits a slight IGC susceptibility, although the base metal exhibits superior IGC resistance. These results indicate that chromium depletion zones were formed near GBs during welding and the IGC resistance decreased even in high-corrosion resistant 316LN stainless steel. Aquino et al. [3] demonstrated the same results for the effect of welding on IGC susceptibility in supermartensitic stainless steel. Iacoviello et al. [4] and Morshed-Behbahani et al. [5] investigated the influence of the heat treatment period on IGC susceptibility in stainless steel using the DL-EPR technique and found that the formation zone of chromium carbide precipitation followed by chromium depletion depends on the heat treatment period, resulting in a change in corrosion behavior. Suresh et al. [6] discussed the IGC resistance of type 304L stainless steel in simulated groundwater based on the carbon content in the steel. They found that high-angle GBs in 304L stainless steel containing more than 0.02% carbon exhibited high IGC susceptibility.

**Figure 1:**
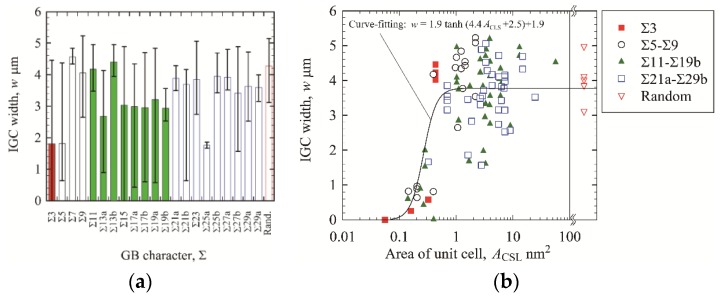
Intergranular corrosion (IGC) susceptibility of grain boundaries (GBs) in sensitized stainless steel (electrochemical potentiokinetic reactivation (EPR) ratio of 29.3%) [21]. (**a**) IGC width as a function of Σ value; (**b**) IGC width as a function of the area of unit cell of coincidence site lattice (CSL) boundaries.

From a microstructural/crystallographic viewpoint, atomic force microscopy (AFM), scanning transmission electron microscopy (STEM), and electron backscattered diffraction (EBSD) techniques have been used to examine the formation of chromium carbide precipitation followed by chromium depletion, and the susceptibility of GBs to IGC. Murr and coworkers [7,8,9,10,11,12] conducted comprehensive studies on the effects of GB misorientation, carbon content, deformation, etc., on the formation behavior of chromium carbide precipitation at GBs in austenitic stainless steel. Liu et al. [13] examined the geometry of corroded grooves via AFM and discussed the relationship between the degree of sensitization (DOS) and the width and depth of the grooves. Bruemmer et al. [14] investigated chromium depletion zones at GBs by energy dispersive X-ray spectroscopy in a TEM and discussed the relationship between the DOS and chromium-depleted zone size. Bi et al. [15] examined the relationship between chromium depletion formation sites and GB structure and found that chromium depletion did not tend to occur at low-energy GBs. Note that GBs have been classified based on their misorientation, namely the difference in crystallographic orientation between adjacent two grains, and the Σ value based on the coincidence site lattice (CSL) model [16]. Srinivasan et al. [17] and Qi et al. [18] discussed the relationship between IGC susceptibility and microstructural features such as grain size, GB structure, and microscopic plastic strain. An et al. [19] investigated the relationship between IGC behavior and GB structure measured by 3D-orientation microscopy consisting of EBSD and serial sectioning techniques, and classified the GB structure into GBs with high and low IGC resistance based on the GB structure. Recently, Haruna et al. [20] pointed out that it is insufficient to characterize the IGC susceptibility of Σ3 boundaries in sensitized austenitic stainless steel determined by EBSD analysis. Fujii et al. [21] also concluded the same result and proposed a parameter (the unit cell area of CSL boundaries) reasonably to characterize the susceptibility of CSL boundaries to IGC. Note that the unit cell area will be explained in Section 2.2 in detail. Figure 1a,b shows the relationships between IGC susceptibility (IGC width) and Σ value and between IGC susceptibility and the unit cell area, respectively. Although the relationship between IGC susceptibility and Σ value exhibited large scatter, the relationship between IGC susceptibility and unit cell area was characterized by a sigmoid curve. The unit cell area is determined based solely on the arrangement of atoms at CSL boundaries, and it is expected that the area is a simple measure that can characterize the IGC susceptibility of CSL boundaries. However, while they demonstrated the IGC susceptibility of type 304 stainless steel with one level of DOS, the applicability of the technique to other DOSs remained to be clarified.

This study aims to elucidate the susceptibility of GBs to IGC in type 304 austenitic stainless steel specimens with various DOSs. IGC testing was conducted under a constant current in oxalic acid, and then a corroded groove formed at the GBs was observed. The crystal orientations of the steel were analyzed via EBSD, and the relationships between the groove size and crystallographic parameters, such as the misorientation, Σ value, and unit cell area of CSL boundaries, were investigated. Especially, the applicability of the evaluation technique using the unit cell area was examined focusing on CSL boundaries.

## 2. Experimental

This section outlines the procedures of specimen preparation and IGC testing used in this study. These procedures are similar to those used in our previous study [21]. See the previous study for details on the testing procedures.

### 2.1. Material

Type 304 austenitic stainless steel was used. Table 1 shows the chemical composition of the steel used. The steel was heated at 1100 °C for 1 h for solutionizing. The average grain diameter of the steel was 119 μm, measured based on Japanese Industry Standard (JIS) G 0551. 

**Figure 2:**
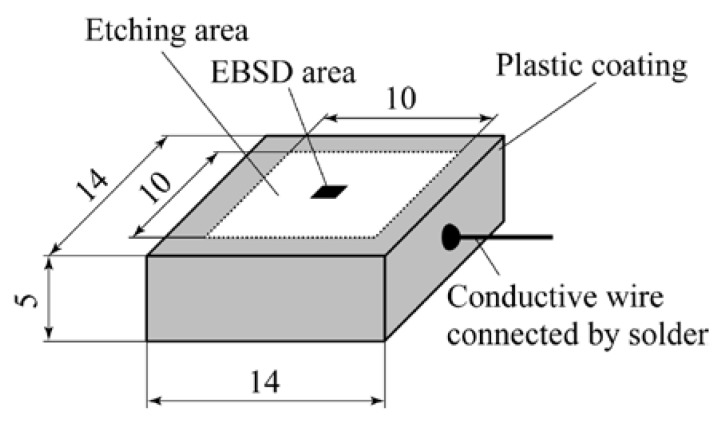
Dimensions of specimens (in units of mm) and preparation for corrosion testing.

**Figure 3:**
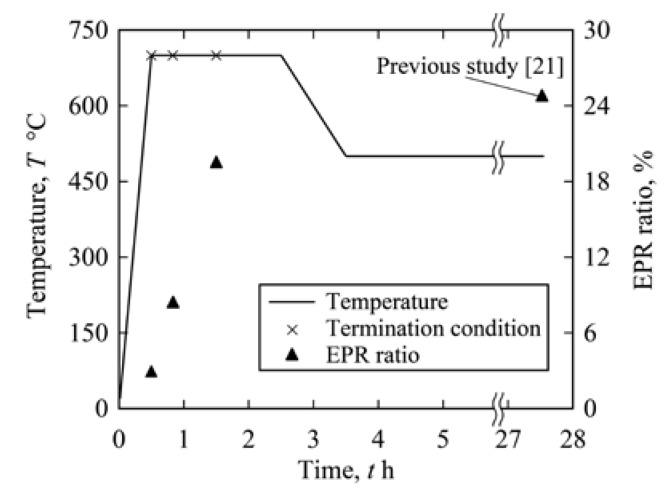
Heating schedule for sensitization and change in EPR ratio of a specimen.

Specimens of 14 × 14 × 5 mm were machined from heat-treated bulk material, as shown in Figure 2. To change the DOS of the specimens, additional heat treatments were applied to the solutionized steel. Figure 3 shows the heating schedule (700 °C for 2 h and 500 °C for 24 h, furnace cooling) used in the previous studies [21,22,23,24,25,26]. The electrochemical potentiokinetic reactivation (EPR) ratio of the steel after the full heating schedule as a measure of DOS was 29.3%, measured by the EPR technique (JIS G 0580). In this study, the heating schedule was terminated after various heating periods to decrease the EPR ratio of the specimen. After terminating the heating schedule, each specimen was furnace-cooled. The EPR ratios of the heat-treated specimens are shown in Figure 3, demonstrating that the EPR ratio was lower for a shorter heating time. 

After the heat treatment, the surface of each specimen was ground with emery paper up to grade #2000 and polished with diamond paste of 3 m grain diameter and colloidal silica (OP-U, Struers, Nagoya, Japan). Then, the surface was ion-milled by argon gas (EM RES101, Leica Microsystems, Wetzlar, Germany) to obtain a smooth and clean surface for EBSD analysis.

### 2.2. Crystallography

Prior to IGC testing, the crystal orientations of a 1 × 1 mm central area in the etching area (black area in Figure 2) were measured with an EBSD system consisting of an orientation imaging microscopy (TSL Solutions, Sagamihara, Kanagawa, Japan). The measurement data were acquired and analyzed by OIM data correction and OIM analysis (TSL Solutions, Sagamihara, Kanagawa, Japan), respectively. Table 2 lists the EBSD measurement conditions. GBs were classified as low-angle (misorientation angle ranging from 5 to 15°) or high-angle (misorientation angle more than 15°), and the high-angle GBs were subclassified as CSL if they exhibited a Σ3–Σ29 structure and random boundaries otherwise. The tolerance angle was used based on the Brandon criterion [27] to determine the CSL boundaries.

**Figure 4:**
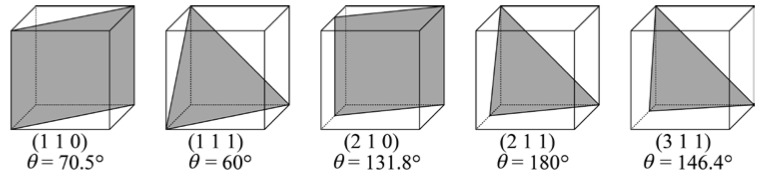
Schematic of GB planes and rotation angles for a Σ3 boundary. *θ* is a misorientation between adjacent grains.

**Figure 5:**
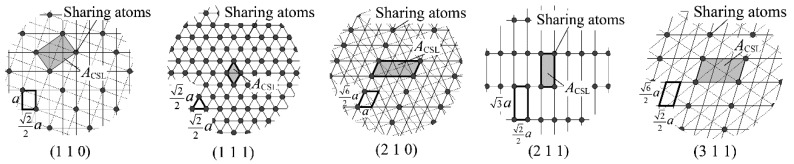
Five kinds of GB structure with atomic configurations for a Σ3 boundary and definition of the area of the CSL unit cell, *A*_CSL_ [21]. *a* is the lattice constant of the steel.

In common EBSD analysis, the structure of a GB at a specimen surface is identified only by the relative orientation relationship between the two adjacent grains. However, Haruna et al. [20] and Fujii et al. [21] pointed out that the analysis yielded an ambiguous result for the GB structure. Each CSL boundary actually exhibits several GB planes (rotation angles and axes). For example, a Σ3 boundary can be formed by the planes {1 1 0}, {1 1 1}, {2 1 0}, {2 1 1}, or {3 1 1}, as shown in Figure 4. We proposed a technique to identify the actual CSL boundary using not only the relative orientation between two adjacent grains, but also the GB trace angle, and applied this technique in this study. To characterize the IGC susceptibility of the CSL boundaries, we used the area of the CSL unit cell, *A*_CSL_. Figure 5 shows the arrangement of atoms on a Σ3 boundary and the technique for determining the value of *A*_CSL_. The solid circles in Figure 5 denote sharing atoms on the boundary, and the value of *A*_CSL_ is defined by the area of the smallest repeating square. The value of *A*_CSL_ is considered to be a measure of the stability of the CSL boundary because the larger the area, the smaller the defects on the CSL boundary. The lattice constant a of the steel used was set to 0.359 nm in this calculation [28]. 

### 2.3. Electrochemical Method for Intergranular IGC Testing 

After EBSD analysis, a conductive wire was connected to the specimen using solder (63/37 Sn-Pb, SE-0ST16, Taiyo Electric Ind. Co., Ltd, Fukuyama, Hiroshima, Japan) the melting temperature of 183–184 °C) and liquid flux (BS-45, Taiyo Electric Ind. Co., Ltd, Fukuyama, Hiroshima, Japan), and all the surfaces except for a 10 × 10 mm etching area were coated with plastic. IGC testing was then conducted based on the oxalic acid etch test for stainless steel (JIS G 0571). The surface was etched in 10% oxalic acid under 1 A/cm^2^ for 90 s with a power source (POLIPOWER, Struers, Nagoya, Japan). After testing, the etched surface was observed under a high-resolution optical microscope (OM) (MS-Z420, Asahi Kogaku Manuf Co. Ltd., Tokyo, Japan). The width of the corroded groove was measured at the midpoint of each GB by the OM, which was regarded as a measure of the susceptibility of the GB to IGC for the sake of simplicity. The GBs for which the corroded groove width was measured were arbitrarily selected in the etched area for each GB class for each specimen.

## 3. Results and Discussion

### 3.1. IGC Behavior

**Figure 6:**
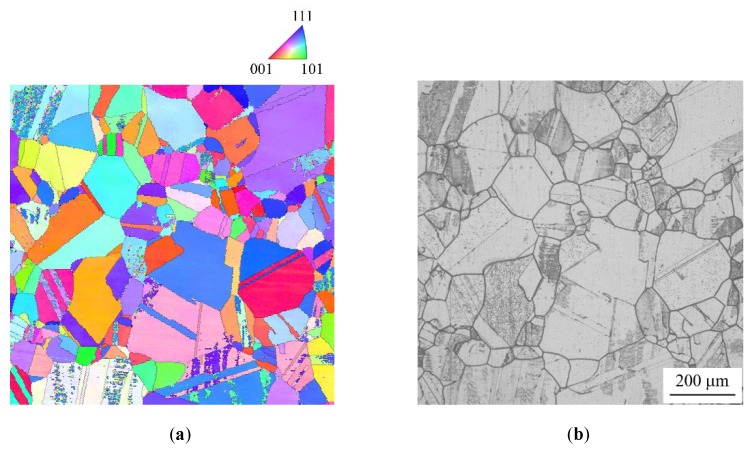
Microstructure of a specimen with high degrees of sensitization (DOS) (EPR of 19.4%). (**a**) Crystal orientation map of specimen surface before etching; (**b**) optical microscope (OM) image of specimen surface etched with oxalic acid corresponding to (a).

**Figure 7:**
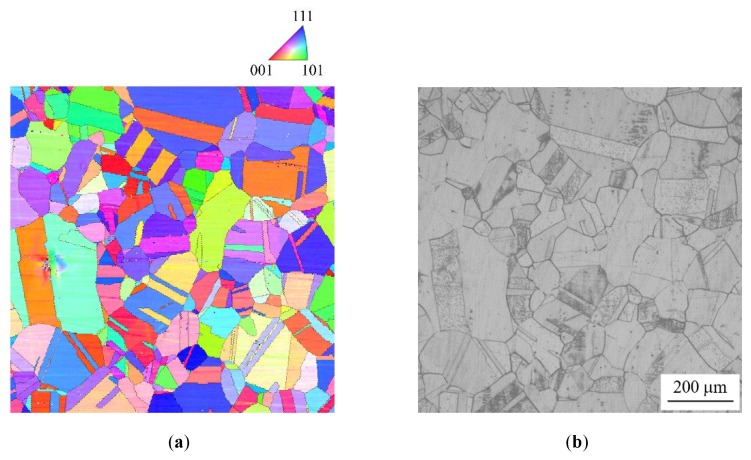
Microstructure of a specimen with low DOS (EPR of 2.8%). (**a**) Crystal orientation map of specimen surface before etching; (**b**) OM image of specimen surface etched with oxalic acid corresponding to (**a**).

Figure 6 and Figure 7 show the crystal orientation maps and IGC profiles of specimens with high DOS (EPR ratio of 19.4%) and low DOS (EPR ratio of 2.8%), respectively. The black lines in Figure 6a and Figure 7a denote GBs with misorientations of more than 5°. In the high DOS specimen, it was confirmed that IGC occurred at some GBs and not at others. In the low DOS specimen, the trend in IGC occurrence was almost the same. The width of IGC in the high DOS specimen seemed to be thicker than that in the low DOS specimen. The relationship between GB structure, EPR ratio, and susceptibility to IGC is discussed in the following sections.

### 3.2. IGC at Low-Angle GBs

**Figure 8:**
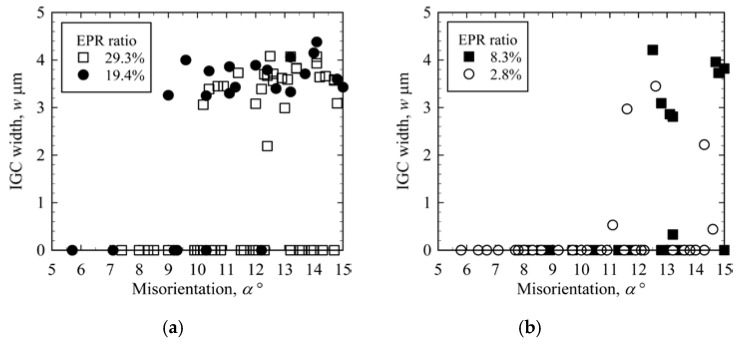
IGC width as a function of misorientation in low-angle GBs. (**a**) High DOS specimens; (**b**) low DOS specimens.

Figure 8 shows the relationship between IGC width and misorientation in low-angle GBs. In high DOS specimens (EPR ratios of 29.3% and 19.4%), IGC did not occur at GBs with a misorientation of less than approximately 10°. For GBs with misorientations of 10–15°, IGC occurred at some of the GBs and not at others, and the IGC width was constant for the corroded GBs. Hence, for high DOS specimens, GBs with misorientations <10° do not exhibit IGC susceptibility and GBs with misorientations of 10–15° may or may not exhibit IGC susceptibility. The trend for the relationship between misorientation and IGC width is the same for low DOS specimens (EPR ratios of 8.3% and 2.8%). However, the critical misorientation shifts rightward and the widths of corroded GBs decrease with decreasing EPR ratio. Moreover, the number of GBs at which no IGC occurred seems to increase with decreasing EPR ratio.

**Figure 9:**
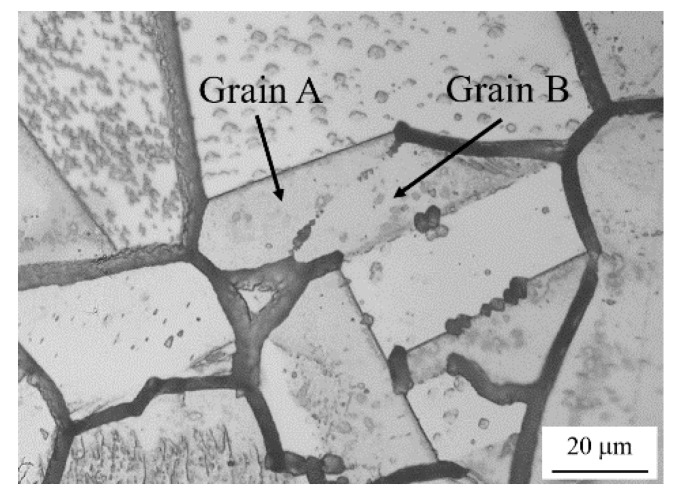
Example of a corroded groove segment at a low-angle GBs in a specimen with an EPR ratio of 19.4%. The misorientation of Grains A and B is 7.1°.

The GBs with misorientations of less than a critical value exhibit little IGC susceptibility and those with misorientations beyond a critical value may or may not exhibit IGC susceptibility, irrespective of the DOS. It is well-known that low-angle GBs have a periodic arrangement of dislocations and the structural stability of low-angle GBs decreases with increasing misorientation between adjacent grains [29]. A chromium depletion zone tends to be formed at GBs with lower structural stability, and the GBs with lower misorientation exhibit little IGC susceptibility. Some of the GBs beyond the critical value exhibit IGC susceptibility, while the others exhibit little IGC susceptibility. This could be explained by the following two reasons. First, the structural stability of GBs is affected not only by the misorientation, but also by the geometric relationship between the GB planes [30]. The formation of chromium carbide followed by a chromium-depleted zone at GBs would depend on their structural stability, resulting in the differences in IGC susceptibility between the GBs. Second, we measured the IGC width at the midpoint of each GB, though the width along the corroded GBs varied. Figure 9 shows an example of a corroded groove at a low-angle GB with a misorientation of 7.1°. Although the IGC width at the midpoint of the GB between Grains A and B in the figure is null, the IGC is several micrometers wide near both ends. It is known that chromium carbide is formed insularly along a GB [31] and a chromium depletion zone would grow from spots along a GB. Hence, as the IGC occurs at the segmentally formed chromium depletion zone along a GB, the IGC susceptibility will vary along a single GB. It would be difficult therefore to characterize in detail the IGC susceptibility distribution along such a GB using a single parameter. Further study related to a measure to evaluate the IGC susceptibility should be conducted.

### 3.3. IGC at High-Angle GBs

**Figure 10:**
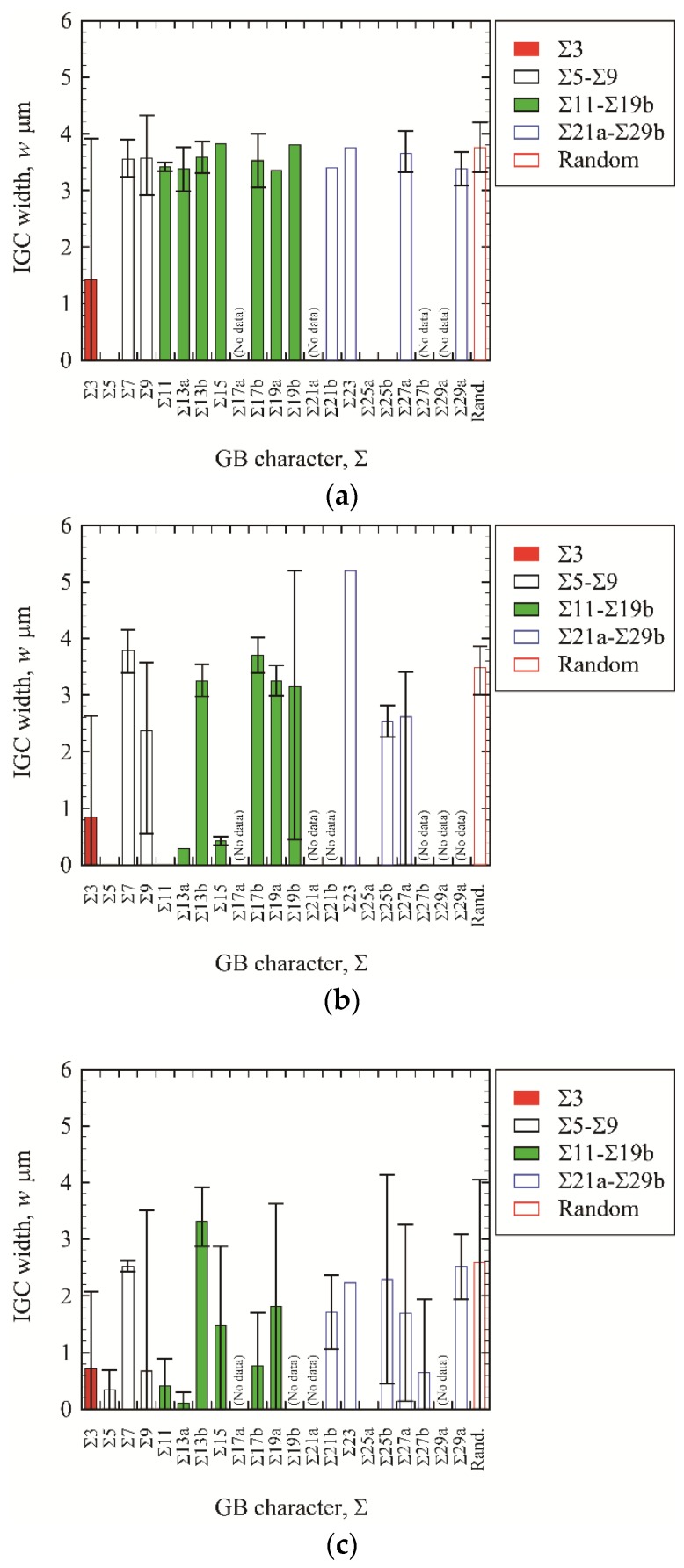
IGC width as a function of value in high-angle GBs. (**a**) EPR ratio of 19.4%; (**b**) EPR ratio of 8.3%; (**c**) EPR ratio of 2.8%.

Figure 10 shows the relationship between IGC width and GB character (Σ value) for high-angle GBs. The scatter bars denote the maximum and minimum widths measured. For the bars with no scatter bar, only one GB was detected in the EBSD-analyzed area. Note that some of the CSL boundaries were not detected in the EBSD-analyzed area, which are denoted as “no data” in this figure.

For the high DOS specimens (EPR ratio: 19.4%), the IGC width drastically fluctuates with increasing Σ value. Additionally, the IGC width for each Σ value exhibits a large scatter. For the low DOS specimens (EPR ratios: 8.3% and 2.8%), the trends are almost the same: the IGC width is unrelated to the Σ value and the IGC width exhibits a large scatter. 

**Figure 11:**
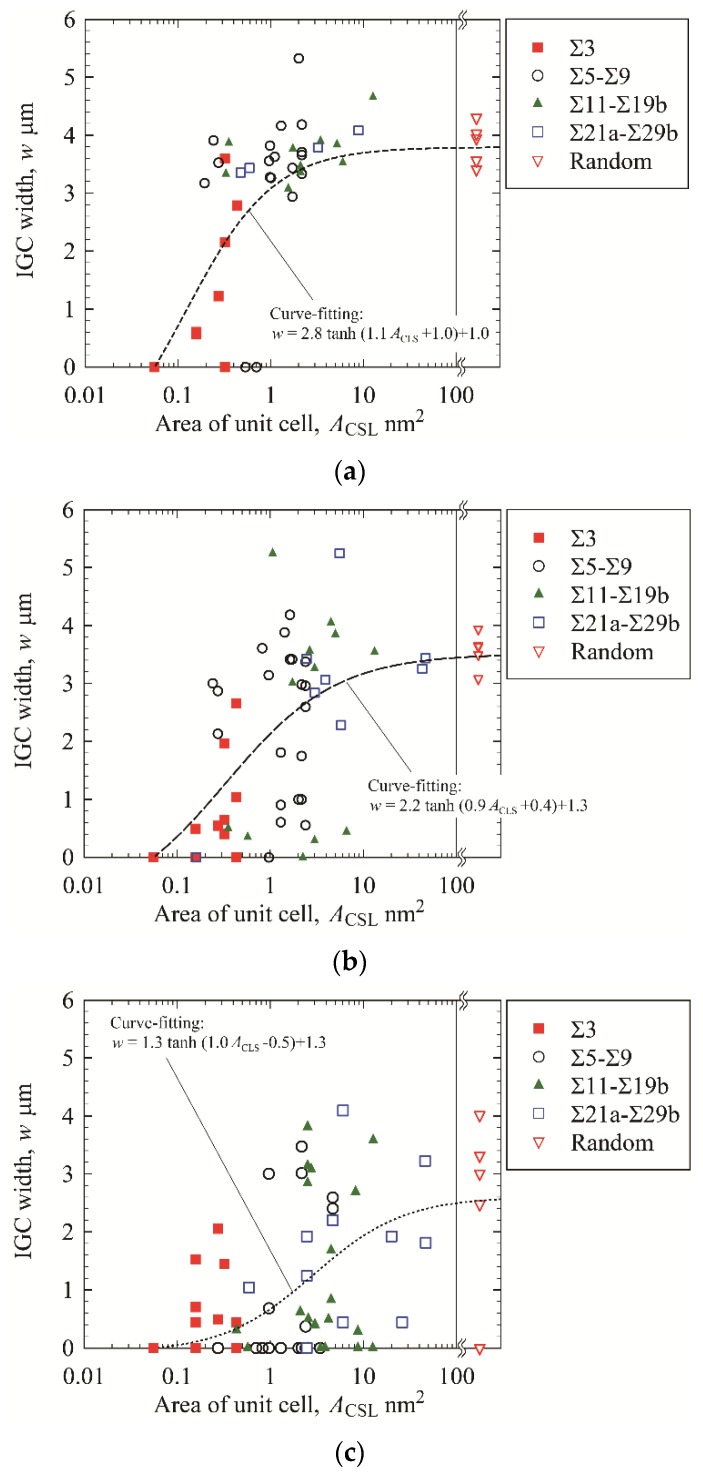
Relationship between IGC width and *A*_CSL_ at GBs. (**a**) EPR ratio of 19.4%; (**b**) EPR ratio of 8.3%; (**c**) EPR ratio of 2.8%.

**Figure 12:**
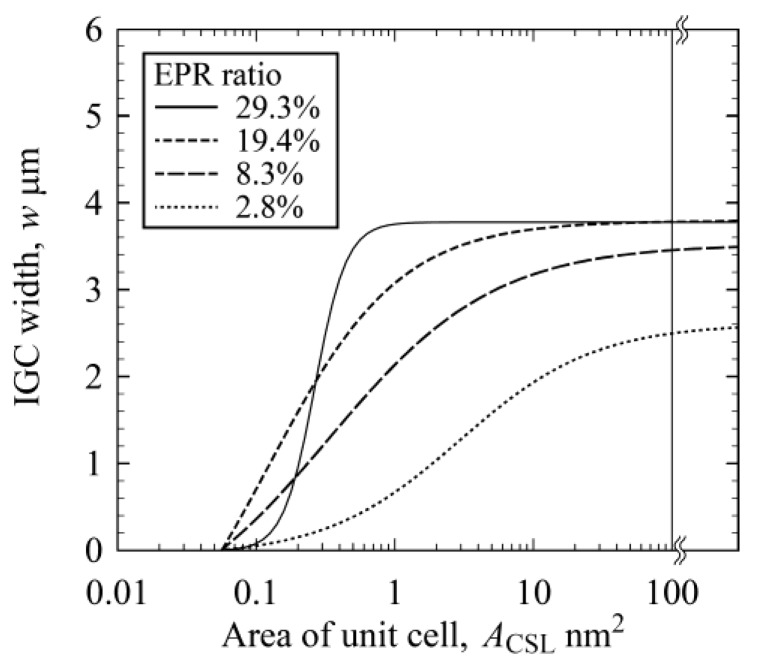
Trendlines of the relationship between IGC width and *A*_CSL_ for all specimens.

Figure 11 shows the IGC width *w* as a function of *A*_CSL_. The IGC widths of the random boundaries are plotted on the right-hand side in the figure because the random boundaries do not have a regular periodic structure and the unit cell cannot be defined. The relationship between IGC width and *A*_CSL_ takes on a sigmoid shape in every case irrespective of the EPR ratio. When *A*_CSL_ is small, no IGC is observed. The IGC width increases with increasing *A*_CSL_ and then levels off. To investigate the influence of the EPR ratio on IGC susceptibility, the trendlines of the relationship between IGC width and unit cell area for all specimens are drawn in Figure 12. Each trendline was obtained by curve-fitting using a hyperbolic tangent function:

(1) $$w=a\tanh\left(bA_{\mathrm{CSL}}+c\right)+d$$

The parameters of *a*, *b*, *c*, and, *d* are obtained using a generalized reduced gradient method to fit the experimental results of each condition. The trendline shifts rightward and the IGC width decreases with decreasing EPR ratio.

**Figure 13:**
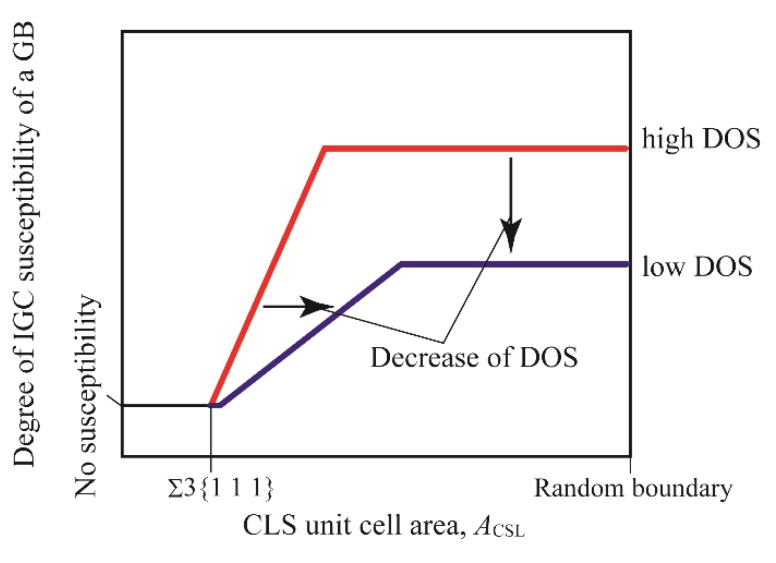
Schematic illustration of the influence of DOS on IGC susceptibility at high-angle GBs. The blue and red lines denote the IGC susceptibility of GBs in high and low DOS specimens, respectively.

The stability is generally evaluated using the Σ value based on the CSL model. The Σ value is defined as the reciprocal of the density of coincidence sites, and it is expected that the stability of CSL boundaries decreases with increasing Σ value. Hence, CSL boundaries with low Σ value may be not sensitized, resulting in a low IGC susceptibility of the boundaries. From Figure 10, however, the IGC width seems not to be dependent on the Σ value, irrespective of the EPR ratio of the specimen. From Figure 11 and Figure 12, on the other hand, the IGC width increases with increasing *A*_CSL_, irrespective of EPR ratio. Consequently, the IGC susceptibility of sensitized stainless steel cannot be characterized using the Σ value, but can be characterized using *A*_CSL_. Figure 13 schematically illustrates the influence of the DOS on IGC susceptibility of high-angle GBs. The relationship between IGC susceptibility and *A*_CSL_ is sigmoid, irrespective of the DOS, and the relationship shifts rightward with decreasing DOS. Romero and Murr [7] found that chromium carbide was formed first at random boundaries, then at incoherent twin boundaries, and then at coherent twin boundaries in type 304 austenitic stainless steel. Suresh et al. [6] and Bi et al. [15] also found that chromium carbide tends to be distributed along high angle random boundaries. These results mean that chromium carbide (followed by a chromium depletion zone) is easily formed at unstable GBs, and the IGC susceptibility increases with decreasing GB structural stability. This study quantitatively expresses this trend of IGC susceptibility using *A*_CSL_ (GB structural stability). In addition, the IGC susceptibility of GBs with large *A*_CSL_ decreases with decreasing DOS. When a specimen is exposed at a high temperature for a shorter period, the chromium depletion zone formation changes and, as for stable GBs such as CSL boundaries with low *A*_CSL_, a smaller chromium depletion zone is formed. For relatively unstable GBs such as random boundaries and CSL boundaries with high *A*_CSL_, the chromium depletion zone is formed in a narrower region in the vicinity of the GBs. As a result, GBs with a larger *A*_CSL_ exhibit less IGC susceptibility, resulting in the low DOS of the specimen. 

The use of *A*_CSL_ is not valid for low-angle GBs and random boundaries because it cannot be defined. A candidate for evaluating the structural stability and IGC susceptibility, irrespective of GB structure, is the GB energy [32,33,34]. The energy can be determined based on the type and arrangement of atoms by numerical methods such as molecular dynamics. However, it is difficult to calculate the energy for each GB in actual conditions. Although some experimental techniques were proposed to measure the GB energy [35,36], the energy could not be determined based on the nondestructive operation: a specimen should be annealed at a high temperature, etc. Hence, it is very difficult to experimentally and/or analytically measure the GB energy of GBs of a material subjected to corrosion. As mentioned before, the unit cell area *A*_CSL_ is simply defined by atom arrangement on CSL boundaries and is easily determined by specimen surface observation via SEM and crystal orientation measurement via EBSD. Although limited to the evaluation of IGC susceptibility at CSL boundaries, this evaluation technique using *A*_CSL_ is a very effective approach. Further investigation of a unified parameter to characterize the susceptibility of low-angle and high-angle GBs is needed.

## 4. Conclusions

In this study, IGC testing was conducted using oxalic acid on thermally sensitized type 304 stainless steel specimens with varied EPR ratios. The susceptibility of GBs to IGC was investigated based on the GB structure, such as their misorientation and Σ value. For low-angle GBs (misorientations ranging from 5° to 15°), GBs with misorientations of less than 10° exhibit little IGC susceptibility, while some GBs with misorientations of 10–15° exhibit IGC susceptibility for high DOS specimens. In high-angle GBs (misorientations >15°), the IGC susceptibility cannot be characterized by the Σ value, but can be characterized by the unit cell area of CSL boundaries, irrespective of the EPR ratio of the steel. The IGC susceptibility increases with increasing unit cell area, and then levels off. The IGC susceptibility of the GBs increases with increasing EPR ratio. To easily characterize the IGC susceptibility of CSL boundaries, the unit cell area of *A*_CSL_, which is simply determined based on atom arrangement at CSL boundaries, is very useful.

## Figures and Tables

**Table 1:** Chemical composition of type 304 austenitic stainless steel (mass %).

C	Si	Mn	P	S	Ni	Cr	Fe
0.06	0.47	0.87	0.03	0.003	8.05	18.16	Bal.

**Table 2:** Conditions of electron backscattered diffraction (EBSD) measurement and analysis using OIM.

Grid Shape	Hexagonal
Measurement interval (m)	3.5
Grain tolerance angle (°)	5
Minimum grain size (pixel)	2
Clean up	Grain dilation, single iteration
